# Treatment Efficacy for Veterans With Posttraumatic Stress Disorder: Latent Class Trajectories of Treatment Response and Their Predictors

**DOI:** 10.1002/jts.22333

**Published:** 2018-10-19

**Authors:** Dominic Murphy, Kirsten V. Smith

**Affiliations:** ^1^ Combat Stress, Research Department Leatherhead United Kingdom; ^2^ King's Centre for Military Health Research King's College London London United Kingdom; ^3^ Oxford Centre for Anxiety Disorders and Trauma, Department of Experimental Psychology University of Oxford Oxford United Kingdom

## Abstract

Evidence suggests that veterans with posttraumatic stress disorder (PTSD) have a poorer treatment response than nonveterans.  In this study, we explored heterogeneity in treatment response for 960 veterans in the United Kingdom with PTSD who had been offered a residential intervention consisting of a mixture of group sessions and individual trauma‐focused cognitive behavioral therapy (TF–CBT). The primary outcome was PTSD score on the Impact of Event Scale–Revised (IES–R).  Covariates included depression, anxiety, anger, alcohol misuse, functional impairment, and sociodemographic characteristics.  Follow‐up occurred posttreatment at set time points for 12 months.  We present predictors of PTSD severity at posttreatment and follow‐up obtained using a latent class growth analysis to identify different treatment trajectories.  Multinomial logistic regression models were used to identify covariates predicting class membership, and five classes were identified. Of participants, 71.3% belonged to three classes showing positive treatment responses, and 1.2% showed initial improvement but later relapsed. Additionally, 27.5% of participants were identified within a treatment‐resistant class that showed little change in severity of presentation. Depression, anxiety, and having had a combat role during military service increased the likelihood of membership in the treatment‐resistant class, odds ratios (*OR*s) = 1.12–1.53, 1.16–1.32, and 2.89, respectively. Additionally, participants in the treatment‐resistant class had higher pretreatment PTSD scores for reexperiencing, avoidance, and hyperarousal symptoms, *OR*s = 5.24, 2.62, and 3.86, respectively. Findings suggest the importance of triaging individuals and offering interventions tailored to severity of presentation.

## Resumen

Spanish Abstracts by Asociación Chilena de Estrés Traumático (ACET)

La Eficacia del Tratamiento para Veteranos del Reino Unido con Trastorno de Estrés Postraumático: Trayectorias de la Clase Latente de la Respuesta al Tratamiento y sus Predictores

TRAYECTORIAS DE TRATAMIENTO PARA LOS VETERANOS DEL REINO UNIDO CON TEPT

La evidencia sugiere que los veteranos con trastorno de estrés postraumático (TEPT) tienen una respuesta al tratamiento más pobre que aquellos que no son veteranos. En este estudio, exploramos la heterogeneidad en la respuesta al tratamiento de 960 veteranos del Reino Unido que presentaban TEPT y a quienes se les había ofrecido una intervención residencial consistente en una mezcla de sesiones grupales y terapia cognitivo‐conductual individual centrada en el trauma (TF‐CBT, en su sigla en inglés). El resultado principal fue el puntaje de TEPT en la Escala Revisada de Impacto de Eventos (IES–R, en su sigla en inglés). Las covariables incluyeron la depresión, la ansiedad, la ira, el abuso de alcohol, la discapacidad funcional, y las características sociodemográficas. El seguimiento ocurrió de forma posterior al termino del tratamiento en periodos específicos por 12 meses. Presentamos predictores de la severidad del TEPT al término el tratamiento y al seguimiento obtenidos por medio de un análisis de crecimiento de clases latentes para identificar las diferentes trayectorias de tratamiento. Modelos de regresión logística multinominales fueron usados para identificar las covariables predictoras de pertenencia a una clase, y así cinco clases fueron identificadas; de los participantes, un 71.3% perteneció a tres clases mostrando respuestas positivas al tratamiento y un 1.2% mostro una mejoría inicial pero posterior recaída. Adicionalmente, el 27.5% de los participantes fueron identificados dentro de una clase resistente al tratamiento, ya que mostraron pocos cambios en la severidad de la presentación. La depresión, odds ratios (*OR*s, en su sigla en inglés) = 1.12 ‐ 1.53; la ansiedad, *OR*s = 1.16 ‐ 1.32; y tener un rol de combate durante el servicio militar, *OR* = 2.89, incrementaron la probabilidad de pertenecer a la clase resistente al tratamiento. Adicionalmente, los participantes en la clase resistente al tratamiento mostraron tener puntajes de más altos de TEPT antes del tratamiento, particularmente reexperimentación, *OR* = 5.24; evitación, *OR* = 2.62; y síntomas de hiperactivación, *OR* = 3.86. Los hallazgos pueden sugerir la importancia de clasificar a los individuos y ofrecer intervenciones a la medida de la severidad de la presentación.

## 抽象

Traditional and Simplified Chinese Abstracts by the Asian Society for Traumatic Stress Studies (AsianSTSS)

簡體及繁體中文撮要由亞洲創傷心理研究學會翻譯

Treatment efficacy for UK veterans with post‐traumatic stress disorder: latent class trajectories of treatment response and their predictors.

Traditional Chinese

標題: 患創傷後壓力症的英國退役軍人的治療成效:治療反應的潛在類別軌跡及其預測變量

撮要: 過往數據證明, 患創傷後壓力症(PTSD)的退役軍人比非退役軍人的治療成效較差。本研究透過960名患PTSD的英國退役軍人, 探索治療成效的異質性。樣本接受住院干預治療, 當中包含組別治療節段和個人的創傷聚焦認知行為治療(TF–CBT)。我們檢視的主要治療成效為於事件影響測量表(修訂版)(IES–R)的PTSD分數。共變量包括抑鬱症、焦慮症、憤怒、濫用酒精、功能性損傷和社會人口特徵。我們在樣本完成治療12個月後作跟進。我們利用潛在類別成長分析, 找出不同的治療軌跡, 並找到於完成治療及跟進期的PTSD嚴重度預測變量。多項邏輯迴歸模型用以找出可預測類別的共變量, 並得出5個類別。71.3%的樣本屬於3個正向治療成效的類別, 1.2%起初展現改善但又回復故態。27.5%的樣本屬於抗拒治療類別, 症狀嚴重度只有少量改變。以下能提升樣本屬抗拒治療類別的可能性:抑鬱症(勝算比 (ORs) = 1.12–1.53)、焦慮症(ORs = 1.16–1.32)、服役時曾參與戰鬥(OR = 2.89)。屬抗拒治療類別的樣本,

治療前以下方面的PTSD分數都較高:再體驗(OR = 5.24)、迴避(OR = 2.62)、過激反應症狀(OR = 3.86)。結果反映把患者分類, 並因應患者的嚴重度調校干預治療的重要性。

标题: 患创伤后压力症的英国退役军人的治疗成效:治疗反应的潜在类别轨迹及其预测变量

撮要: 过往数据证明, 患创伤后压力症(PTSD)的退役军人比非退役军人的治疗成效较差。本研究透过960名患PTSD的英国退役军人, 探索治疗成效的异质性。样本接受住院干预治疗, 当中包含组别治疗节段和个人的创伤聚焦认知行为治疗(TF–CBT)。我们检视的主要治疗成效为于事件影响测量表(修订版)(IES–R)的PTSD分数。共变量包括抑郁症、焦虑症、愤怒、滥用酒精、功能性损伤和社会人口特征。我们在样本完成治疗12个月后作跟进。我们利用潜在类别成长分析, 找出不同的治疗轨迹, 并找到于完成治疗及跟进期的PTSD严重度预测变量。多项逻辑回归模型用以找出可预测类别的共变量, 并得出5个类别。71.3%的样本属于3个正向治疗成效的类别, 1.2%起初展现改善但又回复故态。27.5%的样本属于抗拒治疗类别, 症状严重度只有少量改变。以下能提升样本属抗拒治疗类别的可能性:抑郁症(胜算比 (ORs) = 1.12–1.53)、焦虑症(ORs = 1.16–1.32)、服役时曾参与战斗(OR = 2.89)。属抗拒治疗类别的样本,

治疗前以下方面的PTSD分数都较高:再体验(OR = 5.24)、回避(OR = 2.62)、过激反应症状(OR = 3.86)。结果反映把患者分类, 并因应患者的严重度调校干预治疗的重要性。

A proportion of individuals who leave the military each year with either have symptoms of posttraumatic stress disorder (PTSD) or will go on to develop these difficulties in later life (Fear et al., 2010; Hoge, Riviere, Wilk, Herrell, & Weather, 2014; Sundin et al., 2014). Studies of U.S. military veterans who had been deployed to the conflicts in Iraq or Afghanistan have observed PTSD prevalence rates of between 12%–20% (Hoge et al., 2004; Milliken, Auchterlonie, & Hoge, 2007). Further, there is evidence that as time passes between the ends of these conflicts, increases in prevalence rates of PTSD are being observed (Cabrera, Hoge, Bliese, Castro, & Messer, 2007; Milliken et al., 2007). To date, similar increases have not been witnessed within veteran populations in the United Kingdom (Fear et al., 2010). However, there is evidence of higher prevalence rates of PTSD within subgroups of the U.K. military, such as individuals in combat roles and reservists. Data from Combat Stress, the largest veteran‐dedicated provider of mental health services in the United Kingdom, suggest a sizeable increase in the number of veterans seeking help for PTSD over recent years (Murphy, Weijers, Palmer, & Busuttil, 2015). It has been estimated that the cost to society as a result of PTSD is over and above that of other military‐related mental health difficulties (Brunello et al., 2017; Francois, Depiegel, Maman, Saragoussi, & Auguier, 2010).

Many research papers have reported the efficacy of PTSD treatment programs for veterans from a range of different countries (Chard, Schumm, Owens, & Cottingham, 2010; Currier, Holland, Drescher, & Elhai, 2014; Forbes, Lewis, Parslow, Hawthorne, & Creamer, 2008; Morland et al., 2014; Murphy, Spencer‐Harper et al., 2016; Richardson et al., 2014). In general, the data suggest that existing programs are effective at reducing the burden of PTSD symptoms. However, despite these clinically relevant reductions, there is often still evidence of a significant burden of symptoms. Traditionally, studies exploring treatment outcomes have employed regression analyses when examining symptom reduction. This method assumes all participants share a single treatment trajectory. Latent class growth analysis (LCGA) does not assume this homogeneity and can be used to identify a number of latent classes with qualitatively distinct treatment trajectories, with different growth parameters estimated for each of the latent classes. Using this method, researchers can examine the clinical correlates of differing responses to treatment, such as predictors of fast and slow treatment response and treatment resistance (Jung & Wickrama, 2008). Two studies of veterans have shown the benefits of applying LCGA: One study in a sample of U.S. veterans treated in a residential setting for symptoms of PTSD (Currier et al., 2014) and another in a sample of Australian veterans treated in outpatient settings (Phelps et al., 2018). In both studies, the authors reported distinct subgroups of treatment response—Currier and colleagues (2014) reported three groups and Phelps and colleagues (2018) reported five groups. In addition, the authors identified specific correlates of treatment class. In the U.S. study, the authors found combat exposure and worse health increased the likelihood of poor treatment trajectories, and in the Australian study, authors reported that higher rates of depression and guilt were associated with worse outcomes. Taken together, these results highlight the utility of this approach in elucidating distinct treatment trajectories and the identifying pathways to recovery or treatment resistance.

A further consideration is evidence suggesting that veterans do not respond as well to treatment for PTSD as do nonveteran groups (Bisson, Roberts, Andrew, Cooper, & Lewis, 2013; Kitchiner, Roberts, Wilcox, & Bisson, 2012). The reasons for this disparity are unclear, but a number of potential factors could partially explain this. For example, pre‐trauma risks may include exposure to high rates of childhood adversity (Iversen et al., 2007). In veteran populations in the United Kingdom and within treatment‐seeking groups of veterans in particular, researchers have observed high rates of preservice adversity and reported associations between childhood adversity and PTSD severity (Iversen et al., 2008). Veterans who have served in conflict zones may have been exposed to multiple traumatic experiences, which could increase the complexity of presentations (Richardson, Pekevski, & Elhai, 2009). Evidence suggests that only a minority of veterans with PTSD are able to engage in treatment, and in those who do engage, it can take significant periods of time to get support (Murphy & Busuttil, 2014). In a study of U.K. veterans, authors found that, on average, it took individuals 11 years to seek support and reported an association between taking longer to seek help, greater severity of mental health presentations, and increased risk of residing in areas deemed to be at higher risk of deprivation (Murphy, Palmer, & Busuttil, 2016). By using LCGA, specific risk factors associated with poor treatment response may potentially be able to be identified.

In the current study, we aimed to explore heterogeneity in treatment response within a sample of U.K. veterans who had completed an evidence‐based standardized residential intervention for PTSD. We used LCGA to assess whether, within the sample, there existed subgroups of individuals whose treatment response trajectories over a 12‐month posttreatment follow‐up period were more similar to each other than to the rest of the sample. Further, we assessed predictors of pre‐ and posttreatment PTSD severity and factors associated with class membership. Predictors under investigation included demographic factors (e.g., age, education, and time to seek help), military factors (branch of service, combat exposure, and number of deployments), mental health comorbidities (depression, anxiety, and anger), functional impairment, and alcohol misuse. We anticipated that findings could be used to inform clinicians, improve the identification of predictors of treatment resistance, and aid service development.

## Method

### Participants

The study utilized clinical data from 960 U.K. military veterans who had completed a standardized 6‐week residential treatment program for PTSD between 2013 and 2016.Veterans were referred to the program from a variety of sources from across the United Kingdom, such as the U.K. National Health Service, veteran charities, and self‐referral, among others. Following referral, veterans were assessed by a psychiatrist for a diagnostic interview. If veterans were diagnosed with PTSD, they then completed a second assessment with a psychologist. At this assessment, the clinician explored details of traumatic experiences and assessed inclusion and exclusion criteria for the program. Veterans had to have completed at least one full day of paid employment within the Armed Forces in the United Kingdom (Dandeker, Wessely, Iversen, & Ross, 2006), received a formal diagnosis of PTSD, and reported motivation to start therapy. At assessment, if individuals were currently taking psychiatric medication, they had to remain on a stable dose throughout the course of therapy. Psychiatrists did not make changes to a patient's medication on admission or during the course of therapy. Exclusion criteria included evidence of significant neurological impairment that would affect an individual's ability to engage in psychological therapy (this did not exclude individuals with mild or moderate brain injuries) or being actively psychotic, actively dependent on alcohol or drugs, or actively suicidal. If there was evidence of any of the last three criteria, additional support was provided, and participants were invited for a review at a later date to see if circumstances had changed.

### Procedure

The PTSD treatment program was delivered by psychologists and offered by a mental health charity in the United Kingdom dedicated to supporting veterans. Psychiatric nurses, occupational therapists, art therapists, support workers, and psychiatrists provided additional support. The program has been manualized, and supervision is used to promote treatment fidelity. The treatment ran for 6 weeks between the hours of 9 a.m. and 5 p.m. on weekdays. A typical day consisted of two 1.5‐hr group sessions. Each week, participants were typically offered three individual therapy sessions. The program was offered to closed groups of between 8 and 10 participants and consisted of a mixture of 55 group sessions and 15 individual therapy sessions. Groups broadly fit within two areas: psychoeducational and symptom‐management groups. Psychoeducational groups included the psychological model of PTSD, information on sleep hygiene, and the principals of cognitive behavior therapy (CBT). Symptom management groups included sessions on behavioral activation for depression, strategies to manage anxiety, the use of grounding objects to manage dissociation, mindfulness, and anger management. In addition, patients were offered six weekly art therapy groups and four sessions led by occupational therapists and aimed at promoting recovery. Participants were offered a minimum of 15 individual 90‐min trauma‐focused CBT (TF–CBT) sessions. In the United Kingdom, TF–CBT is approved as the gold standard treatment for PTSD sufferers. There is robust evidence that TF–CBT outperforms other treatment modalities for veterans with PTSD (Bisson et al., 2007; Bisson et al., 2013; Kitchiner et al., 2012; NICE, 2005). Like many therapies for PTSD, in vivo exposure is integral to TF–CBT. A clinician activates trauma memories by asking the patient to bring to mind their traumatic event; the patient is then helped to contextualize the perceptual sensory aspects of the “hot spots,” or worst moments, of the trauma by introducing relevant safety cues and new information about the meaning of the trauma (Ehlers & Clark, 2000; Grey, Young, & Holmes, 2002). This process is called “updating the trauma memory,” and is done through imaginal reliving and verbal processing (Ehlers, Hackmann, & Michael, 2004). Concurrently, patients are encouraged to reengage with avoided stimuli and situations, with a view to extinguishing fearful responding and building mastery. Following the 6‐week program, participants were discharged, and we were unable to monitor what support they may have received from other service providers.

Previously published literature gives a fuller description of the treatment program and demonstrates high rates of treatment completion (94.1%), the lack of a response bias when following up with participants posttreatment, and, in smaller sample sizes, treatment efficacy at 6 and 12 months posttreatment (Murphy, Hodgman et al., 2015; Murphy, Spencer‐Harper et al., 2016).

Data for the study were collected as part of a routine clinical audit. Participants gave consent for data to be used and completed a pack of psychometric measures at the start of therapy (i.e., pretreatment), the end of treatment (i.e., posttreatment), and then three follow‐up time points (6 weeks, 6 months, and 12 months after the end of treatment). A three‐wave postal strategy to elicit responses was used to carry out follow‐up. Ethical approval for this study was provided by the Combat Stress research committee.

### Measures

This was an opportunistic study that took advantage of routinely collected clinical data. As such, only total scores for each measure were recorded, preventing us from reporting of coefficient alpha values. However, the measures used are well‐validated within veteran population studies.

#### Sociodemographic characteristics

At pretreatment, data were collected on a range of sociodemographic factors and details of military service. Sociodemographic variables included sex, age, and level of education, and military service factors included number of deployments, role while on deployment (combat vs. noncombat), service branch (Army, Royal Navy, Royal Marines, or Royal Air Force), and the date the participant left the military. We constructed a new variable using the date of referral to calculate time between leaving the military and starting the treatment program. In addition, a number of psychometric measures were used to assess health and functioning.

#### PTSD symptoms

PTSD was assessed using the 22‐item measure revised Impact of Event Scale (IES–R; Creamer, Bell, & Failla, 2003). The IES–R is informed by the PTSD criteria outlined in the fourth edition of the *Diagnostic and Statistical Manual for Mental Disorders* (*DSM‐IV*). Each item asks participants to rate the frequency with which they have experienced different symptoms of PTSD over the last 7 days on a 5‐point scale ranging from 0 (*not at all*) to 4 (*extremely*). Items are scored from 0 to 4, and total scores can range from 0 to 88, with higher scores suggestive of great severity of symptoms. The scale showed excellent internal consistency (Cronbach's α = .96; Creamer et al., 2003).

#### Depression

Depression was assessed using the nine‐item Patient Health Questionnaire (PHQ‐9; Kroenke & Spitzer, 2002), on which individuals are asked to rate the frequency they experience items related to depression using a 4‐point scale of 0 (*not at all*) to 3 (*nearly every day*). Meeting case criteria was defined as a score of 10 or more on the PHQ‐9. Internal reliability has been shown to be excellent, with reported Cronbach's alpha values between .86 and .89 (Kroenke, Spitzer, & Williams, 2001).

#### Anxiety

Anxiety was assessed using the seven‐item Generalized Anxiety Disorder questionnaire (GAD–7; Swinson, 2006). Meeting case criteria was defined as a score of 8 or above out of a possible score range of 0–21 on the GAD–7. The measure has been shown to have high internal consistency (Cronbach's α = .89; Swinson, 2006).

#### Anger

The five‐item Dimensions of Anger Reactions (DAR‐5; Forbes et al., 2014) was used to assess for problems with anger. Meeting case criteria was defined as a score of 12 or more out of a possible score of 25 on the DAR‐5. Good internal consistency has been reported (Cronbach's α = .88; Forbes et al., 2014).

#### Functional impairment

The Work and Social Adjustment Scale (WSAS; Mundt, Marks, Shear, & Greist, 2002) was used to examine functional impairment. The WSAS explores self‐reported functional impairment across five domains (work, home management, social activities, leisure activities, and close relationships). Scores range from 0 to 40. Cronbach's alpha values for internal scale consistency have been reported to range from .70 to .94 (Mundt et al., 2002).

#### Alcohol misuse

Alcohol misuse was assessed using the Alcohol Use Disorders Identification Test (AUDIT; Babor, Higgins‐Biddle, Saunders, & Monteiro, 2001). Meeting case criteria was defined as a score of 8 or more out of a possible 40 total points on the AUDIT. Cronbach's alpha values ranging from .75 to .94 have been reported (Allen, Litten, Fertig, & Babor, 1997).

### Data Analysis

We conducted analyses using Mplus (Version 7; Muthén & Muthén, 2007). To minimize the bias associated with attrition and missing data, we used the full information maximum likelihood (FIML) approach implemented in Mplus to estimate missing data. Data were found to be missing at random (Little's Missing Completely at Random [MCAR] test), χ^2^(65, *N* = 933) = 69.0, *p* = .343, meeting criteria for maximum likelihood estimation. A majority of participants had data for at least three time points (70.3%); nine participants were excluded because they had data missing at all five time points. Covariance coverage, which measures the impact of missing data, ranged from 0.97 to 0.25 for each pair of variables; this is above the minimum threshold of 0.10 for model convergence (Muthén, 2017).

A conventional latent growth model (LGM), in which intercept and slope vary across individuals and are modeled by random effects, assumes that one intercept and one slope adequately describe a single population with common parameters. Latent growth mixture modeling (LGMM) relaxes this assumption by allowing for differences in growth parameters across unobserved subpopulations or classes (Jung & Wickrama, 2008). Latent class growth analysis (LCGA) is a special case of GMM in which growth trajectories within a class are assumed to be homogenous by fixing the variance of the growth parameters within classes to zero. To aid with model convergence, we chose LCGA as the analysis method for this study. To determine the appropriate class solution, we examined a variety of fit statistics. In particular, the Bayesian (BIC), sample‐size adjusted Bayesian (SSBIC), and Aikaike (AIC) information criterion indices, entropy values, the Lo–Mendell–Rubin likelihood ratio test (LRT), and the bootstrap likelihood ratio test (BLRT; Lo, Mendell, & Rubin, 2001). We sought a model with lower values for the criterion indices, higher entropy values, and significant *p* values for both the LRT and the BLRT. Fit indices in combination with theoretical interpretably guided the final model selection. The LCA models were estimated using robust maximum likelihood method with 1,000 initial stage random starts and 120 final stage optimizations to determine if the best log‐likelihood value was obtained and replicated. Finally, 100 bootstrap draws were used in the BLRT.

A number of covariates were included in the model based on previous research: baseline psychopathology (PTSD and IES–R), anxiety (GAD–7), depression (PHQ‐9), overall functional impairment (WSAS), anger (DAR‐5), and substance abuse (AUDIT) as well as demographics and deployment characteristics including age, level of education (0 = General Certificate of Secondary Education [GCSE] or left school before age 16 years and 1 = A‐level or higher), years between leaving service and starting treatment, number of deployments, service type (0 = Navy, Royal Air Force, or Marines and 1 = Army), and combat exposure (0 = noncombat or combat support role and 1 = combat role). We conducted multinomial logistic regression using SPSS (Version 21) to determine which covariates significantly predicted class membership. Predictors of class membership were entered using a backwards elimination method to avoid suppressor effects due to multicollinearity and ‐2 log‐likelihood thresholds for entering and leaving the model were set to *p* < .05 so that only variables that statistically improved how well the model predicted the outcome variable were retained (Field, 2013). Odds ratios (*OR*) of less than 1 indicate that as a covariate increases, individuals are less likely to be in the comparison class and more likely to be in the reference class.

## Results

### Bivariate Analyses

Descriptive statistics and corresponding zero‐order correlations are presented in Table 1. The sample consisted of 960 veterans with a mean age of 42.99 years (*SD* = 10.61). Participants had been deployed an average of 1.71 times, and 85.9% of the sample were in the Army as opposed to the naval services and the Royal Air Force. In addition to PTSD, 90.4% of participants met case criteria for depression, 94.6% for generalized anxiety, 50.8% for problems with anger, and 45.5% for alcohol problems. These were broadly in line with the characteristics of the wider population of individuals who seek support from Combat Stress (Murphy, Ashwick, Palmer, & Busuttil, 2017). However, of this wider population, only individuals who met the inclusion and exclusion criteria were referred for treatment on the 6‐week treatment program for PTSD, and bed space was limited to approximately 300 participants per year.

**Table 1:** Zero‐Order Correlations of Demographics, Service Characteristics, Baseline Psychopathology, and Posttraumatic Stress Disorder (PTSD) Symptoms Over Time

			PTSD Score (IES–R)				
Variable	*M*	*SD*	Pretreatment	Posttreatment	6‐Week Follow‐Up	6‐Month Follow‐Up	12‐Month Follow‐Up
Age (years)	42.99	10.61	.00	−.01	−.03	.02	.11*
Number of deployments	1.71	1.01	−.04	−.02	−.02	.09	.05
Combat exposure	0.75	0.44	.12***	.06	.09*	.04	.07
Service type	0.84	0.37	.08*	.06	.08*	.05	.00
Time to seek help (years)	13.04	10.74	.03	.04	−.01	.04	.11*
Level of education	0.25	0.43	−.11***	−.06	−.07	−.08	−.11
Depression (PHQ)	17.12	5.22	.62***	.34***	.42***	.39***	.39***
Anxiety (GAD‐7)	15.51	4.23	.60***	.27***	.38***	.36***	.38***
Anger (DAR)	11.55	4.56	.33***	.17***	.17***	.19***	.18***
Overall functioning	25.73	8.11	.33***	.18***	.27***	.31***	.24***
Alcohol (Audit)	8.63	8.37	.07*	.03	.02	.03	.06

*Note*. IES–R = Impact of Event Scale, Revised; PHQ = Patient Health Questionnaire; GAD = Generalized Anxiety Disorder 7‐Item scale; DAR = Dimensions of Anger Reactions scale; AUDIT = Alcohol Use Disorders Identification Test.

*p < .05. ***p < .001.

Service type and combat exposure were significantly associated with PTSD at baseline and 6‐week follow‐up, indicating that veterans who served in the Army or in a combat role had higher PTSD scores before treatment and at 6‐week follow up compared to those in other branches of the armed forces with noncombat roles. Being older at assessment was associated with having more PTSD symptoms at 12‐month follow‐up but not significantly associated with PTSD symptoms any other time point. A higher level of education was associated with fewer PTSD symptoms at baseline, but there was no association between education and PTSD at any other time point. Taking longer to seek treatment after leaving the service was associated with higher PTSD symptoms at 12‐month follow‐up. All measures of baseline psychopathology except alcohol use were significantly associated with PTSD in the expected direction at all five time points. Alcohol use at baseline was associated with a significantly higher level of PTSD symptoms at baseline only. Only psychopathology and background variables significantly associated with PTSD symptom severity at one time point or more were included in the covariate class analysis.

### Simple Growth Models

Using the likelihood ratio chi‐square test to determine model fit, we examined models with an intercept parameter (no growth); intercept and slope parameters (linear growth); and intercept, slope, quadratic, and cubic parameters (both nonlinear growth). The linear model provided a significant improvement in fit over the intercept‐only model for PTSD symptoms, indicating an overall pattern of change for PTSD symptoms across time. The quadratic effect demonstrated superior fit over the linear growth curve, and the cubic growth curve demonstrated superior fit compared to the quadratic model.

### Latent Class Growth Analysis Findings

**Figure 1:**
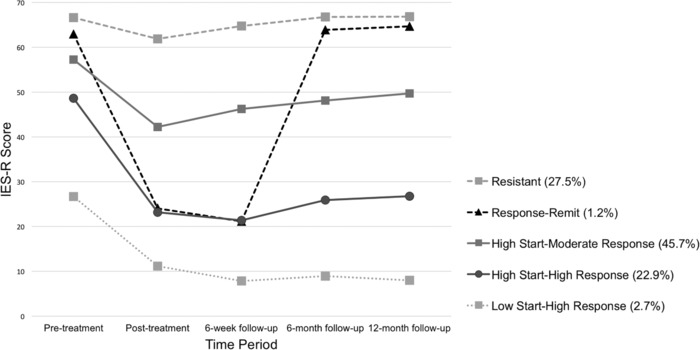
Five‐class latent growth analysis of Impact of Events Scale–Revised (IES–R) posttraumatic stress disorder scores from pretreatment to 12‐month follow‐up.

A comparison of Models 1–6 suggested that a five‐class model provided the best fit to the data (Table 2) on the majority of the metrics (Wickrama, Lee, O'Neal, & Lorenz, 2016). The AIC and SSBIC, but not the BIC, were lower when the class solution moved from four to five classes, and entropy was highest in the five‐class solution. The BLRT suggested that the five‐class solution was superior to the four‐class solution, but the VLMR‐LRT was just short of significance, *p =* .064. However, in simulation studies, the BLRT has been shown to consistently outperform the VLMR‐LRT in correctly identifying the optimal class solution (Nylund, Asparouhov, & Muthén, 2007). Furthermore, findings from a recent study showed that the SSBIC identified the correct class solution with higher frequency than other indicators and demonstrated that the BIC performed less well in the presence of a smaller number of classes (Morgan, 2015).This was the case with Class 1 in the five‐class model, which represented 1.2% of the total population. Overall, these results, taken in context with class size, point to a five‐class solution (see Figure 1). Class‐specific estimates are shown in Table 3. Class 1 was defined as having a “response–remit trajectory” (1.2% of the sample). Class 2 was the second smallest class, holding 2.7% of the sample; veterans with this trajectory exhibited a “low start–high response” to treatment. Class 3 was the second largest class, with 27.5% of the sample, and was classified as individuals with a “resistant” trajectory over time. Class 4, “high start–high response,” was characteristic of the trajectory of 22.9% of the sample. Class 5 was the largest class and included 45.7% of the sample; individuals with membership in this class had a trajectory classified as “high start–moderate response” to treatment.

**Table 2:** Fit Indices for Latent Class Growth Analysis Examining Posttraumatic Stress Symptoms From Baseline to 12‐Month Follow‐Up

Number of Classes	AIC	BIC	SSBIC	Entropy	VLMR‐LRT *(p*)	BLRT (*p*)
1 class	27,850	27,874	27,858	–	–	–
2 class	26,978	27,026	26,994	0.71	< .001	< .001
3 class	26,771	26,844	26,796	0.63	< .001	< .001
4 class	26,728	26,825	26,762	0.67	< .001	< .001
5 class	26,708	26,830	26,750	0.69	.064	< .001
6 class	26,700	26,846	26,751	0.61	.659	.667

*Note. N* = 960. AIC = Aikaike information criterion; BIC = Bayesian information criterion; SSBIC = sample‐size adjusted Bayesian information criterion; VLMR‐LRT = Lo–Mendell–Rubin likelihood ratio test; BLRT = bootstrap likelihood ratio test.

**Table 3:** Class‐Specific Parameter Estimates for the Five‐Class Solution

		Intercept			Slope			Quadratic			Cubic		
Class	%^a^	Est.	*SE*	*p*	Est.	*SE*	*p*	Est.	*SE*	*p*	Est.	*SE*	*p*
Response–remit	1.2	61.24	2.62	< .001	−70.25	21.42	.001	29.93	8.00	< .001	−2.95	0.73	< .001
Low start–high response	2.7	29.11	4.37	< .001	−25.67	5.38	< .001	8.57	1.85	< .001	−0.078	0.17	< .001
Resistant	27.5	65.99	0.81	< .001	−4.75	1.57	.003	2.16	0.63	.001	−0.22	0.07	.001
High start–high response	22.9	47.03	1.12	< .001	−39.79	2.89	< .001	13.92	1.11	< .001	−1.28	0.11	< .001
High start–moderate response	47.5	55.68	1.02	< .001	−18.64	1.86	< .001	6.87	0.88	< .001	−0.64	0.10	< .001

*Note. N =* 960. Est. = parameter estimates.

a Percentage of participants with class membership.

### Covariates of Class Membership

Of the predictor variables we investigated, only depression, *p* < .001; anxiety, *p* < .001; and combat exposure, *p* = .021, made unique contributions and therefore remained in the model. Results indicated that having a higher PHQ–9 or GAD–7 score at admission was significantly predictive of whether or not a participant would respond to and maintain gains in treatment. As depression scores increased, so did the likelihood that veterans would be in the resistant group compared to the high start–moderate response group, *OR* = 0.90; the high start–high response group, *OR* = 0.82; and the low start–high response group, *OR* = 0.85 (Table 4). For baseline depression scores, the resistant group did not differ from the response–remit group. The same pattern was observed for baseline anxiety; as anxiety increased, veterans were more likely to be in the resistant group than the high start–moderate response group, *OR* = 0.87; the high start–high response group, *OR* = 0.84; and the low start–high response group, *OR* = 0.76. However, the resistant group did not differ from the response–remit group. Individuals in a combat role were 2.86 times more likely to be in the treatment resistant group compared with the low start–low response group.

**Table 4:** Multinomial Logistic Regression Predicting Class Membership for the Five‐Class Model

	PHQ			GAD			Combat Exposure		
	β	*SE*	*OR*	β	*SE*	*OR*	β	*SE*	*OR*
Response–remit vs. Resistant	−.07	.10	0.93	−.17	.12	0.85	.94	1.1	2.57
Low start–high response vs. Resistant	−.43	.06	0.65***	−.28	.07	0.76***	−1.10	.49	0.35*
High start–high response vs. Resistant	−.19	.03	0.82***	−.18	.04	0.84***	−.28	.24	0.76
High start–moderate response vs. Resistant	−.11	.03	0.90***	−.14	.03	0.87***	.20	.21	1.22

*Note*. PHQ = Patient Health Questionnaire; GAD = Generalized Anxiety Disorder questionnaire; OR = odds ratio.

*p < .05. **p < .01. ***p< .001.

### PTSD Symptom Cluster Analyses

We ran three further multinomial logistic regression analyses to determine whether the magnitude of specific cluster symptoms on the IES–R (i.e., reexperiencing, avoidance, and hyperarousal) were predictive of class membership. While all three clusters inherently make up class membership, we were interested in the relative differences in beta values between the three clusters as a metric of clinical interest (i.e., does the resistant class present with higher reexperiencing symptoms in comparison to a class that starts with a similar overall symptom severity, such as the high start–moderate response class or the response–remit class?). The resistant group did not differ from the response–remit class in terms of any of the three symptom clusters. However, the resistant group did differ significantly from the high start–moderate response class on all three clusters. The likelihood of being in the resistant class versus the high start–moderate response class was 5.24 times more likely, *p* < .001, as reexperiencing symptoms increased; 3.86 times more likely, *p* < .001, as hyperarousal symptoms increased; and 2.62 times more likely, *p* < .001, as avoidance symptoms increased.

## Discussion

In this study, we explored treatment response to a standardized residential intervention for veterans with PTSD. The intervention was offered over a 6‐week period and consisted of a mixture of group therapy and individual TF–CBT. Follow‐ups were conducted at set time points over 12 months posttreatment. In this study, we explored three key areas: factors associated with a higher burden of PTSD symptoms at both (a) pretreatment and (b) 12 months posttreatment as well as (c) the use of latent class growth analysis to assess for the presence of different treatment trajectories.

At pretreatment, several factors were associated with higher PTSD scores and, hence, greater PTSD severity. Preservice factors included reporting a lower level of educational achievement, and factors related to military service included having served with the Army or having a combat role. Several comorbid health difficulties were also associated with more serious pretreatment PTSD, including higher scores on measures of depression, generalized anxiety, difficulties with anger, and alcohol misuse. In addition, higher self‐reported levels of functional impairment were also associated with a greater burden of PTSD symptoms.

At 1‐year follow‐up, higher rates of depression, anxiety, and anger were still associated with a higher burden of PTSD symptoms. Alcohol misuse was no longer significantly associated with higher PTSD scores, implying that even though alcohol misuse may predict more serious pretreatment PTSD presentations, alcohol misuse, at least in this sample, did not interfere with positive treatment outcomes. This suggests that it may be possible to successfully treat veterans for PTSD alongside dual‐diagnosis alcohol problems. Both older age and taking longer to seek help after leaving the military were associated with higher PTSD scores at 12‐month follow‐up. Taken together, it is possible that individuals who have taken longer to seek help may have more entrenched PTSD symptoms. Although the reasons for this are unknown, it could be that experiencing symptoms of PTSD for longer periods of time leads to increased erosion of resources, such as social support and employment opportunities, for individuals. Some evidence that may support this theory comes from studies of mental health presentations of veterans that have observed associations between taking longer to seek support and an increased risk of experiencing multiple deprivation (Currier et al., 2014; Murphy, Palmer, & Ashwick, 2017).

We identified five classes of treatment trajectories. Classes were named according to their PTSD symptom trajectories. These included three groups that showed a positive treatment response, which were classed as low start–high response, high start–high response, and high start–moderate responses. Of participants, 71.3% were assigned to these classes. There were 27.5% of participants who were assigned to a treatment‐resistant class. A minority of participants (1.2%) belonged to a response–remit class. In this class, significant reductions in PTSD scores were observed posttreatment, and then, at the first follow‐up point 6 weeks later and at subsequent timepoints, PTSD severity scores returned to pretreatment levels. Class membership was associated with depression, anxiety, and having a combat role during military service, with higher depression and anxiety scores increasing the likelihood of being in the treatment‐resistant class. Figure 1 suggests that initial PTSD severity may indicate class membership. These findings fit with those reported in previous research from a variety of countries that has identified psychological comorbidities as predictors of poor treatment response (Currier et al., 2014; Murphy & Busuttil, 2015; Richardson, Eihai, & Sareen, 2011). Analysis of the IES–R symptom cluster subscales indicated that reexperiencing symptoms was the strongest predictor of membership in the treatment‐resistant class.

The present data appear to support moving away from a “one‐size‐fits‐all” intervention for PTSD and on to tailoring interventions based upon presentation. Evidence from a sample of Australian veterans engaged in treatment for PTSD who were offered either residential settings, outpatient clinics, or a mixture of the two suggested that for individuals with mild PTSD presentations, it may be more prudent to provide less resource‐intensive interventions, such as outpatient support (Creamer, Forbes, Biddle, & Elliot, 2002). Further, there is a growing body of evidence demonstrating the efficacy of trauma therapies delivered remotely via the Internet (Turgoose, Ashwick, & Murphy, 2017). These approaches may be less intrusive for veterans and more cost‐effective for clinical services. Data from the current study could be interpreted as supportive of these findings and of the need to triage veterans. For example, individuals with mild PTSD presentations may not have needed residential treatment, whereas residential treatment was indicated for individuals with moderate or higher severity presentations.

The current study explored the heterogeneity within treatment response and found a subgroup of treatment resistant veterans. The presence of this subgroup of veterans with more complex presentations may explain the disparity in treatment response between veterans and nonveterans. These findings strongly suggest the importance of developing new interventions to support this subgroup. Further, although several factors have been reported both within this study and in previously published research, further research of currently recommended interventions is needed to more accurately identify veterans at risk of treatment resistance.

Authors of previous research have shown the most serious presentations of PTSD in veterans in the United Kingdom are associated with higher levels of comorbid mental health difficulties, substance misuse, childhood adversity, and not being in a relationship, all of which may be indicative of more complex presentations (Murphy et al., 2017). The 11th revision of the *International Classification of Diseases* (*ICD‐11*; World Health Organization, 2018) was published in 2018 and includes a diagnosis of complex PTSD; it seems prudent to explore this diagnosis within veteran populations. In the current study, we found that higher rates of depression and anxiety increased the likelihood of treatment resistance. This highlights the importance of treating comorbid mental health difficulties both prior to when veterans engage in trauma‐focused therapy and also during the course of therapy.

This study profited from a large sample size, long‐term follow‐up, and previous research within this population that indicated the absence of a response bias in participants with whom follow‐up was successful. However, there are several limitations that need to be considered when interpreting the data. First, we employed an observational design in the study that took advantage of routinely collected clinical data. Although this may improve the representativeness of the sample, as they were recruited directly from a national clinical service, it does mean that there was no control group or randomization to control for confounders. For example, it could be that the improvements in PTSD symptoms observed within this sample resulted from natural recovery and were not related to treatment. However, given the chronicity of presentations (on average 11 years) and long‐term follow‐up, it seems unlikely that the improvement in symptoms resulted from spontaneous improvements.

Second, the finding that most veterans with PTSD showed improvement posttreatment is restricted to the intervention having been offered in a residential setting and consisting of a mixture of group therapy and individual sessions of TF–CBT. This also means that it is difficult to identify the active ingredients of the 6‐week intervention as it was not possible to explore the individual components of the intervention (e.g.,TF–CBT sessions or group sessions). However, it seems likely that the TF–CBT was the key element as the efficacy of TF–CBT in the treatment of veterans with PTSD has been reported previously (Bisson et al., 2013). As such, findings can't be generalized to outpatient settings or in situations when TF–CBT was delivered in isolation. In addition, we only had access to total scores for each outcome measure, which meant that more detailed analysis was not possible.

Third, it was not possible to explore other factors, such as type of combat exposure, core emotions association with trauma memories (e.g., shame‐based vs. fear‐based memories), number of traumatic experiences, or the presence of childhood adversity, which could be hypothesized from clinical experience as predictive of treatment response. Future researchers may wish to explore the impact of these factors on treatment response, as this could be used to more accurately predict which subgroups of veterans are at risk of not responding to current treatments and may thus need new, novel interventions. The use of interventions specifically developed to treat emotional deregulation and interpersonal difficulties (both of which are associated with complex PTSD), such as dialectical behavior therapy coupled with prolonged exposure (Harned, Korslund, & Linehan, 2014) or Skills Training in Affective Interpersonal Regulation (STAIR; Cloitre & Schmidt, 2015), may be useful to support individuals with the most complicated needs.

Fourth, we were unable to access information on psychiatric medication use other than the need for medication to remain stable during the 6‐week treatment period or information about what additional support may have been offered to participants by other organizations following completion of the 6‐week treatment program. This needs to be considered when interpreting the findings as changes in medication posttreatment or the receipt of additional support may have impacted on treatment trajectories.

Finally, the measures used to explore treatment outcomes were reliant on self‐report. Although the use of self‐report measures is common research evaluating treatment outcomes, the additional use of a diagnostic assessment with a clinician could have added to a better understanding of class response.

This study presents evidence that the majority of veterans with PTSD showed improvements in the severity of their PTSD scores 12 months after treatment. However, a sizeable minority did not respond to treatment. Having a combat role during military service and comorbid depression and anxiety increased the likelihood of treatment‐resistance. The findings of the current study suggest that more accurate triaging to assess severity of presentations would be helpful to ensure better targeted care and that more research is needed to better support the needs of the most vulnerable veterans. It could be that this group may have had more serious presentations of PTSD that may fit with the new *ICD‐11* diagnosis of complex PTSD, although more research is needed to confirm this.
